# Magnetic Properties of the Ferromagnetic Shape Memory Alloys Ni_50+*x*_Mn_27−*x*_Ga_23_ in Magnetic Fields

**DOI:** 10.3390/ma7053715

**Published:** 2014-05-08

**Authors:** Takuo Sakon, Kohei Otsuka, Junpei Matsubayashi, Yuushi Watanabe, Hironori Nishihara, Kenta Sasaki, Satoshi Yamashita, Rie Y. Umetsu, Hiroyuki Nojiri, Takeshi Kanomata

**Affiliations:** 1 Department of Mechanical and Systems Engineering, Faculty of Science and Technology, Ryukoku University, Otsu 520-2194, Japan; E-Mails: t100180@mail.ryukoku.ac.jp (K.O.); t100249@mail.ryukoku.ac.jp (J.M.); t100270@mail.ryukoku.ac.jp (Y.W.); h_nishi_hara@yahoo.co.jp (H.N.); 2 Department of Mechanical Engineering, Graduate School of Engineering and Resource Science, Akita University, Akita 010-8502, Japan; E-Mail: saza6694@yahoo.co.jp; 3 Faculty of Engineering, Tohoku Gakuin University, Tagajo 985-8537, Japan; E-Mail: s.y.0641247@gmail.com; 4 Institute for Materials Research, Tohoku University, Sendai 980-8577, Japan; E-Mails: rieume@imr.tohoku.ac.jp (R.Y.U.); nojiri@imr.tohoku.ac.jp (H.N.); 5 Research Institute for Engineering and Technology, Tohoku Gakuin University, Tagajo 985-8537, Japan; E-Mail: kanomata@tjcc.tohoku-gakuin.ac.jp; 6 Department of Materials Research, Graduate School of Engineering, Tohoku University, Sendai 980-8579, Japan

**Keywords:** shape memory alloys, thermal strain, magnetization, magnetic properties, magnetic field, itinerant electron ferromagnet

## Abstract

Thermal strain, permeability, and magnetization measurements of the ferromagnetic shape memory alloys Ni_50+*x*_Mn_27−*x*_Ga_23_ (*x* = 2.0, 2.5, 2.7) were performed. For *x* = 2.7, in which the martensite transition and the ferromagnetic transition occur at the same temperature, the martensite transition starting temperature *T*_Ms_ shift in magnetic fields around a zero magnetic field was estimated to be d*T*_Ms_/d*B* = 1.1 ± 0.2 K/T, thus indicating that magnetic fields influences martensite transition. We discussed the itinerant electron magnetism of *x* = 2.0 and 2.5. As for *x* = 2.5, the *M*^4^ *vs. B/M* plot crosses the origin of the coordinate axis at the Curie temperature, and the plot indicates a good linear relation behavior around the Curie temperature. The result is in agreement with the theory by Takahashi, concerning itinerant electron ferromagnets.

## 1. Introduction

Ferromagnetic shape memory alloys have been extensively studied as potential candidates for smart materials. Among these alloys, Ni_2_MnGa is the most familiar alloy [1]. It has a cubic *L*2_1_ Heusler structure (space group $Fm\;\bar{3}\;m$) with the lattice parameter *a* = 5.825 Å at room temperature, and it orders ferromagnetically at the Curie temperature *T*_C_ ≈ 365 K [2,3]. Upon cooling from room temperature, a martensite transition occurs at the martensite transition temperature *T*_M_ ≈ 200 K. Below *T*_M_, a superstructure forms because of lattice modulation [4,5]. For Ni-Mn-Ga Heusler alloys, the *T*_M_ can vary from 200 to 330 K by nonstoichiometrically changing the concentration of composite elements.

Several studies on Ni-Mn-Ga alloys address the martensite transition and the correlation between magnetism and crystallographic structures [6–18]. Ma *et al.* [7] studied the crystallography of Ni_50+*x*_Mn_25_Ga_25−*x*_ alloys (2 ≤ *x* ≤ 11) by powder X-ray diffraction and optical microspectroscopy. In the martensite phase, typical microstructures were observed for *x* < 7. The martensite variants exhibit configurations typical of self-accommodation arrangements. The TEM image of Ni_54_Mn_25_Ga_21_ indicates that the typical width of a variant is about 1 μm.

Umetsu *et al.* [19] made a phase diagram of Ni_50+*x*_Mn_27−*x*_Ga_23_ alloys (−25 ≤ *x* ≤ 6). The martensite transition temperature *T*_M_ and the ferromagnetic transition temperature (Curie temperature) *T*_C_ cross over at around *x* = 2.5. The martensite transition temperature of Ni_50+*x*_Mn_27−*x*_Ga_23_ is higher than that of the stoichiometric composition. This property is very useful from the viewpoint of developing commercial applications.

The interaction between magnetism and crystallographic rearrangements was discussed in references [1,8,17,18]. Murray *et al.* [18] studied polycrystalline Ni-Mn-Ga alloys. The magnetization step at *T*_M_ is also observed. This is a reflection of the magnetic anisotropy in the tetragonal martensite phase. In the martensite phase, a strong magnetic anisotropy exists. Under these circumstances, the magnetization that reflects the percentage of magnetic moments parallel to the magnetic field is smaller than that of the austenite phase, where the magnetic anisotropy is not strong in the weak magnetic field. Therefore, the magnetization step is observed at *T*_M_.

NMR experiments indicate Mn-Mn indirect exchange via the faults in Mn-Ga layers interchange, which are caused by excessive amounts of Ga [13]. This result indicates that the exchange interaction between Mn-Mn magnetic moments is sensitive to lattice transformation: during such a transformation, the magnetism changes from that of a soft magnet in the austenite phase to that of a hard magnet in the martensite phase; this is due to higher magnetic anisotropy. Dai *et al.* [20] reported this softening of the lattice; they did so by measuring the elastic constants of a Ni_0.50_Mn_0.284_Ga_0.216_ single crystal using the ultrasonic continuous-wave method. *C*_11_, *C*_33_, *C*_66_ and *C*_44_ modes were investigated; every mode indicated an abrupt softening around *T*_M_. This lattice softening appears to be affected by the abrupt expansion just above *T*_M_ when cooling from the austenite phase.

Takahashi proposed a spin fluctuation theory of itinerant electron magnetism [21,22]. The induced magnetization *M* is written as the formula of

$${\left(\frac{M}{M_{S}}\right)}^{4}=1.20\times 10^{6}\frac{T_{C}^{2}}{T_{A}^{3}p_{S}^{4}}\cdot \frac{H}{M}$$ (1)

where $M_{S}=N_{0}\mu_{B}p_{S}$ is the spontaneous magnetization in the ground state; *N*_0_ is the molecular number; $p_{S}=gS$, where *g* is the g-factor and *S* is a spin; *T*_A_ is the spin fluctuation parameter. Nishihara *et al.* [23] measured the magnetization of Ni and Ni_2_MnGa. Good linearity is observed in the *M*^4^ *vs. H/M* plot at the Curie temperature for Ni. This result indicates that the critical index δ (defined as $H\propto M^{\delta }$) is 5.0.

In this study, we focused on the physical effects of magnetic fields. By using polycrystalline samples, it was possible for us to provide information concerning the easy axis of magnetization in a martensite structure; we made temperature-dependent strain measurements under constant magnetic fields. In this paper, permeability, thermal strain, and magnetization measurements were performed for polycrystalline Ni_50+*x*_Mn_27−*x*_Ga_23_ (*x* = 2.5, 2.7) in magnetic fields (*B*). Thermal strain and magnetization results of Ni_52_Mn_25_Ga_23_ (*x* = 2.0) were used for discussing the magnetic field dependence of the martensite transition temperature and magnetization [24]. The results of thermal strain in a magnetic field and magnetic-field-induced strain yield information about the twin boundary motion in the fields. The experimental results were compared with those of other Ni-Mn-Ga single crystalline or polycrystalline alloys, and correlations between magnetism and martensite transition were found. Itinerant magnetism is also discussed, based upon the results of the magnetic field dependence of the magnetization and compared with the predictions of Takahashi’s theory [21,22].

The martensite transition temperature *T*_M_ and reverse martensite phase transition *T*_R_ are used when these are quoted from the references. To define these temperatures clearly, we used *T*_M_ and *T*_R_ as the martensite transition starting temperature *T*_Ms_ and the reverse martensite temperature finishing temperature *T*_Rf_ in our research results.

## 2. Experimental Procedures

The Ni_50+*x*_Mn_27−*x*_Ga_23_ (*x* = =2.5, 2.7) alloys were prepared by arc melting 99.99% pure Ni, 99.99% pure Mn, and 99.9999% pure Ga in an argon atmosphere. To obtain homogenized samples, the reaction products were sealed in double-evacuated silica tubes, and then annealed at 1123 K for 3 days and quenched in cold water. The obtained samples were polycrystalline. From X-ray powder diffraction, the monoclinic *6M* phase martensite structure and the *D0*_22_ tetragonal structure were mixed at 298 K. The size of the sample was 2.0 mm × 2.0 mm × 4.0 mm.

The measurements in this study were performed at atmospheric pressure (*P* = 0.10 MPa). Thermal strain measurements were performed using strain gauges (Kyowa Dengyo Co., Ltd., Chofu, Japan). The electrical resistivity of the strain gauges was measured by the four-probe method. The strain gauge was fixed parallel to the longitudinal axis of the sample.

Thermal strain measurements were performed using a 10 T helium-free cryo-cooled superconducting magnet at the High Field Laboratory for Superconducting Materials, Institute for Materials Research, Tohoku University. The magnetic field was applied along the sample’s longitudinal axis. The thermal strain is denoted by the reference strain at 360 K.

Magnetization measurements in a steady field at 5K and temperature dependence of the magnetization at 0.10 T were performed using a SQUID magnetometer installed at Ryukoku University. As for *x* = 2.7, the high temperature magnetization between 320 K and 370 K were performed using a home-made magnetometer within a weak AC fields (with the frequency *f* = 73 Hz and the maximum field *B*_max_ = 0.0050 T, which has a compensating high homogeneity magnetic field. AC fields were applied along the sample’s longitudinal axis.), using an AC wave generator WF 1945B (NF Co., Ltd., Yokohama, Japan) and an audio amp PM17 (Marantz Co. Ltd., Kawasaki, Japan). The magnet was the same magnet with the thermal strain measurements. Pulsed magnetization measurements were performed using a Bitter-type water-cooled pulsed magnet (inner bore: 26 mm; total length: 200 mm) in a pulsed magnetic field at Ryukoku University. The magnetic field was applied along the sample’s longitudinal axis. The values of magnetization were corrected using the values of spontaneous magnetization for 99.99% pure Ni. The magnetic permeability measurements were performed in AC fields, which is as same as the AC magnetometer. Thermal experiments were carried out by means of DSC, using a rate of 10 K/min.

## 3. Results and Discussion

### 3.1. Magnetic Properties of Ni_52.5_Mn_24.5_Ga_23_ (x = 2.5)

**Figure 1.**
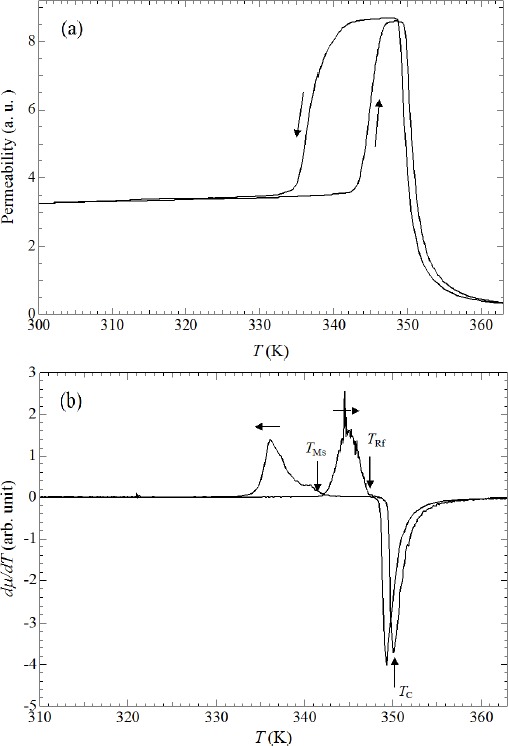
(**a**) Temperature dependence of the magnetic permeability μ of Ni_52.5_Mn_24.5_Ga_23_ in AC fields with *f* = 73 Hz and *B*_max_ = 0.0050 T. The origin of the vertical axis is the reference point when the sample is empty in the pickup coil of the magnetic permeability measurement system; (**b**) the *d*μ/*dT vs. T. T*_Ms_ and *T*_Rf_, which correspond to the characteristic temperature of martensite transition for thermal strain shown in Figure 2, are indicated by arrows.

Figure 1a shows the temperature dependence of permeability. When heating from 300 K, permeability increases gradually. As shown in Figure 1a, permeability increases above 343 K and suddenly decreases around 350 K. When cooling from a high temperature, permeability shows a sudden increase at 355 K and decreases below 342 K. The sudden changes in permeability indicate that the ferrromagnetic transition occurs around 350 K. The temperature dependence of permeability for Ni_52.5_Mn_24.5_Ga_23_ is similar to that of Ni_52_Mn_12.5_Fe_12.5_Ga_23_’s, which shows a transition of a ferromagnetic–martensite (Ferro–M) phase to a ferromagnetic–austenite (Ferro–A) phase [25]. The step above 343 K (heating process) and below 342 K (cooling process) reflects stronger magnetic anisotropy in the tetragonal martensite phase, compared with that in the cubic austenite phase [8,18,24].

**Figure 2.**
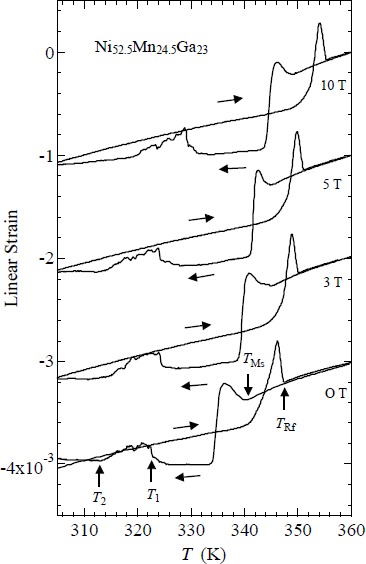
Temperature dependence of the linear thermal strain of Ni_52.5_Mn_24.5_Ga_23_ in static magnetic fields.

Polycrystalline Ni_49.5_Mn_28.5_Ga_22_, Ni_50_Mn_28_Ga_22_, and Ni_52_Mn_12.5_Fe_12.5_Ga_23_ alloys also indicate the magnetization (or permeability) step at *T*_M_ [9,18,26] below the field of 10 mT. Figure 1b indicates the temperature dependence of the differential of permeability *d*μ*/dT*. The martensite transition starting temperature *T*_Ms_ and reverse martensite finishing temperature *T*_Rf_, which correspond to the characteristic temperature of martensite transition for thermal strain shown in Figure 2, are indicated by arrows.

Figure 2 shows the linear thermal strain of Ni_52.5_Mn_24.5_Ga_23_. *B* means magnetic field and the unit is T (tesla). *B* is equal to μ_0_*H*, where μ_0_ is the absolute permeability of a vacuum; the unit of *H* is A/m. At zero magnetic fields, the memory strain was observed, as polycrystalline Ni_53.6_Mn_27.1_Ga_19.3_ [10]. When heating from 300 K, a slight strain was observed, first at zero magnetic fields. Above 342 K, a sharp strain is observed. The results of previous studies [6,7] suggest that this is because of the reverse martensite transition *T*_Rf_ = 347 K, which is denoted by an arrow. When cooling from 360 K, a sudden decrease is observed at 341 K. The martensite transition starting temperature *T*_Ms_ is 341 K, defined as the midpoint temperature of the transition. The permeability at the Ferro–M phase is very low compared with that of the Ferro–A phase. The results of permeability and linear strain measurements indicate that the region above *T*_Ms_ is a Ferro–A phase and that below *T*_Ms_ is a Ferro–M phase. The permeability measurement results indicate that the ferromagnetic transition from the paramagnetic–austenite (Para–A) phase to the Ferro–A phase occurs around *T*_C_ = 350 K (see Figure 1a). At zero magnetic fields, and in magnetic fields, there is no visible anomaly around *T*_C_. When cooling from 360 K, the thermal strain also shows a peak at 336 K. This may be attributed to the intermingling of the *L*2_1_ austenite lattices and the 6*M* martensite lattices at the martensite transition. A sequential phenomenon is observed in single crystalline Ni_2.19_Mn_0.81_Ga [20]. The thermal strain shows an anomaly around *T*_1_ = 323 K and *T*_2_ = 313 K. The reason of this anomaly is open question at the present time. The contraction at *T*_Ms_ under zero field is about 0.8 × 10^−3^ (0.08%). As for other Heusler alloys, Ni_52_Mn_12.5_Fe_12.5_Ga_23_ and Ni_2_Mn_0.75_Cu_0.25_Ga, the contraction occurs at the martensite temperature [26]. The strain at *T*_M_ of polycrystalline Ni_52_Mn_12.5_Fe_12.5_Ga_23_ was estimated as 0.14% contraction. This value is larger than that of Ni_52.5_Mn_24.5_Ga_23_. After zero field measurements of the linear strain, measurements in magnetic fields from 3 T to 10 T were performed. The strains at *T*_Ms_ under the magnetic field were estimated as 0.08% contraction, which is the same as that at zero magnetic field (0.08%).

**Figure 3.**
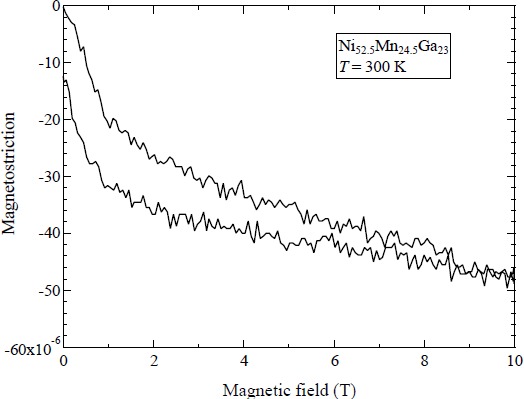
Magnetostriction of Ni_52.5_Mn_24.5_Ga_23_ at 300 K in a static magnetic field of up to 10 T.

Figure 3 shows the magnetic-field-induced strain at 300 K (Ferro–M phase) in a static magnetic field. When increasing the magnetic field from zero field, a sudden contraction occurs up to 1 T. Above 1 T, a gradual contraction is observed. When decreasing the magnetic field from 10 T, a modicum of strain occurs. Below 1 T, a sudden strain is observed. The magnetic-field-induced strain at 10 T is −50 ppm or −0.005%.

The reasons that magnetic-field-induced strain is smaller than the strain at *T*_M_, in the linear strain measurements, were elucidated in references [18,24,27].

On heating from the martensite phase, an abrupt increase occurred in the field-induced strain around *T*_M_. They suggest that this is caused by lattice softening near *T*_M_. As for the thermal strain of Ni_52.5_Mn_24.5_Ga_23_, shown in Figure 2, peaks appear for both *T*_Ms_ and *T*_Rf_ in zero field and all values of the magnetic field. The peak at *T*_Rf_, which is associated with heating, is larger than the one at *T*_Ms_, which is associated with cooling. These peaks indicate that the lattice expands abruptly. The ultrasonic continuous-wave measurements by Dai *et al.* [20] indicated abrupt softening around *T*_M_. This lattice softening appears to be affected by the abrupt expansion just above *T*_M_ or *T*_R_, when cooling or heating from or to the austenite phase, respectively.

**Figure 4.**
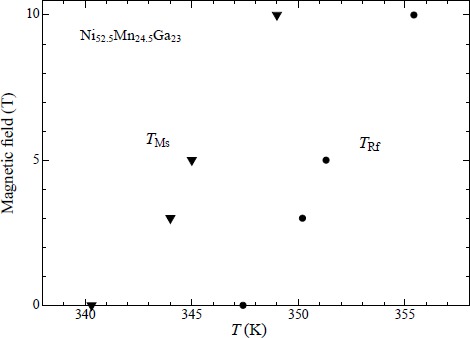
Magnetic phase diagram of Ni_52.5_Mn_24.5_Ga_23_. Filled triangles indicate the martensite transition start temperature *T*_Ms_. Filled circles indicate the reverse martensite finishing temperature *T*_Rf_.

Figure 4 shows the magnetic phase diagram of Ni_52.5_Mn_24.5_Ga_23_. With increasing field, *T*_Ms_ and *T*_Rf_ gradually increase. The shifts in *T*_Ms_ and *T*_Rf_ around zero magnetic field are estimated as d*T*_Ms_/d*B* = 0.9 ± 0.2 K/T and d*T*_Rf_/d*B* = 0.8 ± 0.2 K/T, which is larger than those of the Ni_52_Mn_12.5_Fe_12.5_Ga_23_ alloy’s (d*T*_M_/d*B* = 0.5 K/T) [26]. In Section 3.2, we will discuss the shifts in *T*_Ms_ and *T*_Rf_ under magnetic fields.

**Figure 5.**
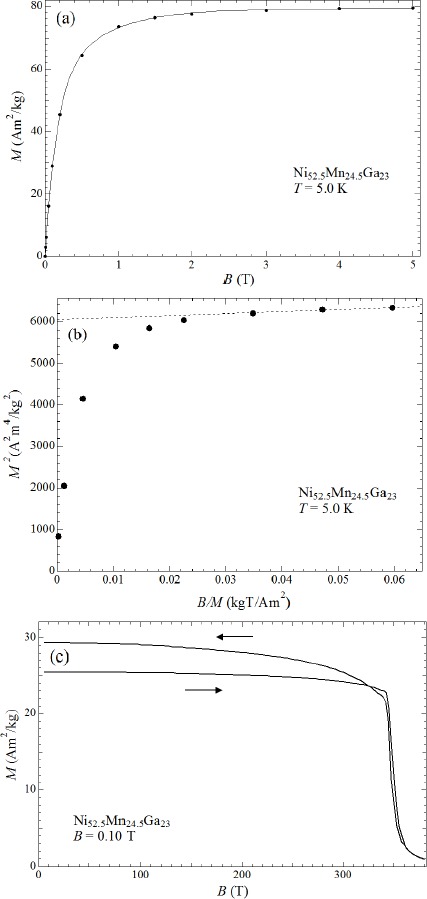
(**a**) Magnetization of Ni_52.5_Mn_24.5_Ga_23_ in a static magnetic field; (**b**) Arrott plot of magnetization at 5 K; (**c**) temperature dependence of magnetization at 0.10 T.

Figure 5a shows the magnetization curves of Ni_52.5_Mn_24.5_Ga_23_ at 5 K in a static magnetic field measured by a SQUID magnetometer. The unit of magnetization *M* is Am^2^/kg in the SI unit system or emu/g in the CGS unit system (both have identical numerical values). The saturation magnetization was 79.6 Am^2^/kg. The spontaneous magnetization was 77.7 Am^2^/kg (which was derived from the Arrott plot) as shown in Figure 5b. This spontaneous magnetization value is equal to a magnetic moment of 3.35 μ_B_/f.u. This absolute value corresponds to 3.75 μ_B_/Mn. The Mn magnetic moment of 3.75 μ_B_ is comparable to the magnetic moment of Mn on the B site, which is approximately 3.58 μ_B_/Mn (as obtained from the powder neutron scattering experiments performed by Ahuja *et al.* [28]). Detailed expressions about the magnetic moments of Ni_50+*x*_Mn_27−*x*_Ga_23_ alloys (−25 ≤ *x* ≤ 6) were described by Umetsu *et al.* [19].

Figure 5c shows the temperature dependence of magnetization at 0.10 T. During the heating process, a decrease in magnetization occurred between 350 and 360 K, which corresponds to the magnetic transition, as shown in Figure 1a. Notably, this abrupt decrease in the *M*-*T* curve at *T*_Ms_ is different from that of usual ferromagnets. The assumed reason is that martensite transition occurs just below the Curie temperature *T*_C_ for Ni_52.5_Mn_24.5_Ga_23_. For *x* =5, the *M*-*T* curve shows a gradual decrease at *T*_Ms_ during the heating process. At this composition, the magnetic transition occurs in the martensite phase.

**Figure 6.**
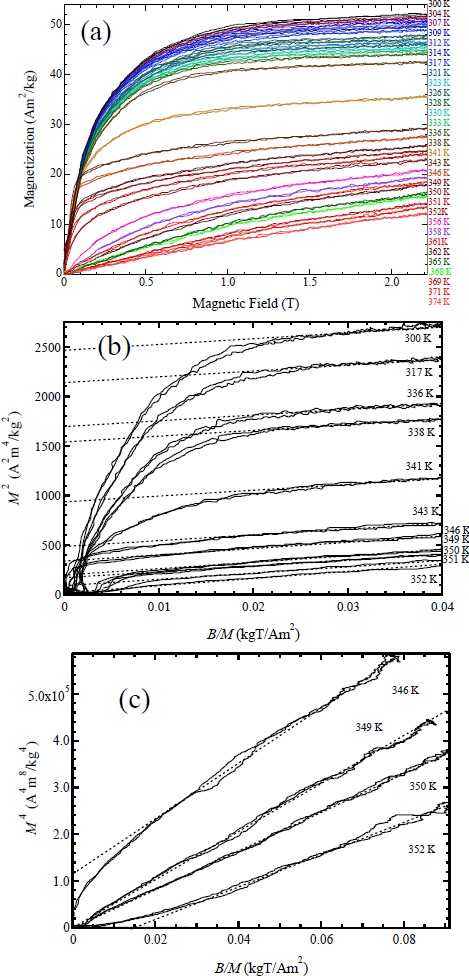
(**a**) Magnetization of Ni_52.5_Mn_24.5_Ga_23_ in a pulsed magnetic field up to 2.2 T; (**b**) Arrott plot of the magnetization of Ni_52.5_Mn_24.5_Ga_23_. Dotted straight lines are extrapolated lines; (**c**) *M*^4^ *vs. B/M* plot for Ni_52.5_Mn_24.5_Ga_23_. Dotted straight lines are extrapolated lines.

Figure 6a shows the magnetization curves of Ni_52.5_Mn_24.5_Ga_23_ in a pulsed magnetic field up to 2.2 T. The *M-B* curves were measured from low temperatures. The hysteresis of the *M-B* curve is considerably small. The magnetocaloric effects in other magnetic materials were also reported; for example, Levitin *et al.* [29] reported for Gd_3_Ga_5_O_12_. They performed magnetization measurements at an initial temperature of 4.2 K, where the magnetic contribution to heat capacity is comparable to that of the lattice heat capacity. In our experiment, the temperature change of the sample due to the magnetocaloric effect is considered to be within 1 K. This is because these experiments were performed around room temperature, where the lattice heat capacity is much larger than the heating or cooling power of the magnetocaloric effect. Moreover, *M-B* curves in Figure 6a show rather small hysteresis, which indicates that the magnetizations have been measured under a static temperature condition.

The *M*-*B* curves show ferromagnetic behavior below 350 K. Clearly, the field dependence of magnetization at the Ferro–A phase above *T*_Rf_ = 347 K is different from that of the Ferro–M phase around *T*_Rf_. At the Ferro–M phase, magnetization increases with magnetic fields. On the other hand, at the Ferro–A phase between 334 and 356 K, a sudden increase in magnetization occurs between 0 and 0.1 T.

Figure 6b shows the Arrott plot of Ni_52.5_Mn_24.5_Ga_23_. This plot was also used for estimating the spontaneous magnetization to discuss the d*T*_M_/d*B* by means of the Clausius-Clapeyron relation. The spontaneous magnetization in Ni_52.5_Mn_24.5_Ga_23_ at 338 K, just below *T*_R_ is 39.1 Am^2^/kg, which was obtained by the Arrott plot in Figure 6b. When using this value as the *M*_S_, the magnetocrystalline anisotropy energy in the martensite phase of Ni_52.5_Mn_24.5_Ga_23_ is *M*_S_*B*_S_/2 = *K*_U_ = 1.7 × 10^5^ J/m^3^, which is on the same order as that in the martensite phase of Ni_2_MnGa. These magnetic properties were also shown for Ni_51.9_Mn_23.2_Ga_24.9_ [11], Ni_49.5_Mn_25.4_Ga_25.1_ [12], and Ni_54_Mn_21_Ga_25_ [13].

At large *B/M* values, the *M*^2^ *vs. B/M* plot seems to show a linear relation. However, around *T*_C_, the *M*^2^ *vs. B/M* plot strays out from a linear relation at low *B/M* values. Therefore, we made an attempt of other plot.

Figure 6c shows the *M*^4^ *vs. B/M* plots. The *M*^4^ *vs. B/M* plot indicates a good linear relation around the Curie temperature *T*_C_ = 350 K. The critical index δ for *M*^δ^ *vs. B* is 5.0. The result is in agreement with the theory by Takahashi for weak itinerant electron ferromagnets [21,22].

**Figure 7.**
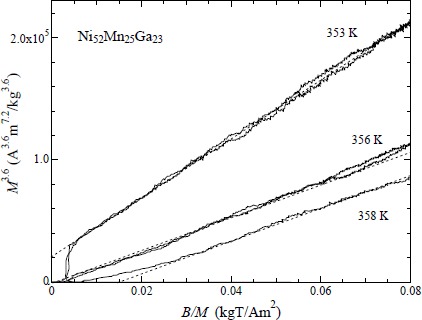
*M*^3.6^ *vs. B/M* plot for Ni_52_Mn_25_Ga_23._ Dotted straight lines are extrapolated lines.

The *M*^3.6^ *vs. B/M* plot for Ni_52_Mn_25_Ga_23_ is shown in Figure 7. The magnetic properties were reported in our former research [24]. This plot indicates a good linear relation around the Curie temperature *T*_C_ = 356 K. The critical index δ is 4.6. Nishihara *et al.* [23] investigated the magnetic field dependence of the magnetization of Ni_2_MnGa around *T*_C_ = 363 K. The critical index δ is 4.77. As for Takahashi theory’s formula as shown in Equation (1), the critical index δ is 5.0. Therefore, we analyze of the characteristic temperature *T*_A_ for Ni_52.5_Mn_24.5_Ga_23_.

From the magnetic moment 3.75 μ_B_/Mn obtained from our magnetization measurement and the gradient of *M*^4^ *vs. B/M* in Figure 6c, we calculated the characteristic temperature *T*_A_ as 1.06 × 10^4^ K. This value is comparable to the value of Co_2_CrGa (1.0 × 10^4^ K) [30] and Ni (1.76 × 10^4^ K) [21]. These results reflect the nature of itinerant magnetism for Ni_52.5_Mn_24.5_Ga_23_.

### 3.2. Magnetic Properties of Ni_52.7_Mn_24.3_Ga_23_ (x = 2.7)

**Figure 8.**
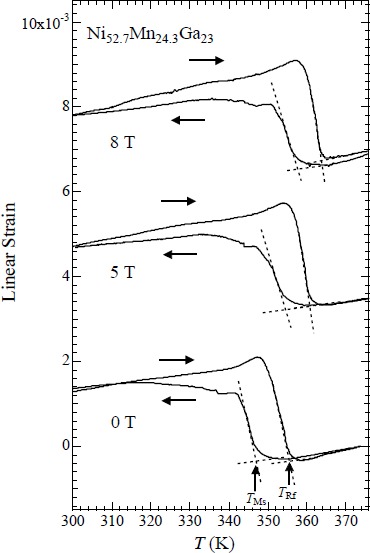
Temperature dependence of the linear thermal strain of Ni_52.7_Mn_24.3_Ga_23_ in static magnetic fields, parallel to the longitudinal direction of the sample.

Ni_52.7_Mn_24.3_Ga_23_ is an alloy in which the martensite transition and ferromagnetic transition occurred at the same temperature [19]. Figure 8 shows the temperature dependence of the linear thermal strain of Ni_52.7_Mn_24.3_Ga_23_ in static magnetic fields, parallel to the longitudinal direction of the sample. With increasing field, *T*_Ms_ and *T*_Rf_ gradually increased. The shifts in *T*_Ms_ and *T*_Rf_ around zero magnetic field are estimated as d*T*_Ms_/d*B* and d*T*_Rf_/d*B* as 1.1 ± 0.2 K/T. Thermal strain results indicate that, with cooling from austenite phase, the elongation occurred at *T*_Ms_. The same phenomenon was observed in the Ni_2.19_Mn_0.81_Ga and Ni_2.20_Mn_0.80_Ga polycrystalline samples. It should be noted that these alloys also show the martensitic and ferromagnetic transitions at the same temperature [31]. For the temperature dependence of the linear thermal strain of Ni_52.7_Mn_24.3_Ga_23_ perpendicular to the longitudinal direction, contraction occurred at *T*_Ms_ with cooling from austenite phase. As for the lattice parameters of Ni_52.7_Mn_24.3_Ga_23_, martensite phase *c*-axis parameter is 11% larger than austenite phase *a*-axis parameter, and, martensite phase *a*-axis parameter is 6% smaller than austenite phase *a*-axis parameter [19]. Therefore, it is conceivable that the difference of these linear strains parallel or perpendicular to the longitudinal direction of the sample is that the sample is oriented to some extent.

**Figure 9.**
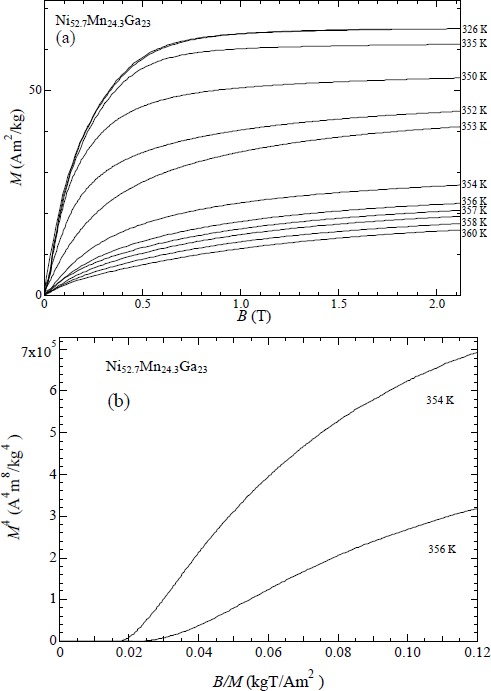
(**a**) Magnetization of Ni_52.5_Mn_24.5_Ga_23_ in a static field; (**b**) *M*^4^ *vs. B/M* plot for Ni_52.7_Mn_24.5_Ga_23_.

Figure 9a shows the magnetization process of Ni_52.7_Mn_24.3_Ga_23_. The experiments were performed in steady fields. This is because that this alloy shows the martensite transition and ferromagnetic transition at the same temperature. Then the latent heat is not small at *T*_C_. In order to avoid heating or cooling due to the ferromagnetic and martensite transition, we measured in steady fields and isothermal processes. The magnetization suddenly changes at *T*_C_ = 356 K. The spontaneous magnetization change ∆*M* is 28 Am^2^/kg. The *M*^4^ *vs. B/M* plot is shown in Figure 9b. This plot did not indicate a linear relation around the Curie temperature. Takahashi’s theory can be applied to isotropic ferromagnet. Below *x* = 2.5, *T*_C_ is higher than *T*_M_ and a ferromagnetic transition occurs in the austenite phase. Between *T*_M_ and *T*_C_, the magnetic property is isotropic ferromagnet. On the contrary, *x* = 2.7 transfers from the martensite ferromagnet to the austenite paramagnet. In the martensite phase, magnetic anisotropy is larger than that in the austenite phase. Therefore, it is supposed that Takahashi’s theory cannot be applied to *x* = 2.7.

### 3.3. The Magnetic Field Dependence of the Martensite Transition Temperature

Now we discuss about the field dependence of the martensite transition temperature.

The martensite transition temperature change (d*T*) induced by magnetic field change (d*B*) is approximately given by the Clausius-Clapeyron relation [8,32,33]:

$$\frac{dB}{dT}=\frac{\Delta S}{\Delta M}\cdot \left[T/K\right]$$ (2)

where, ∆*S* and ∆*M* are the differences in entropy and magnetization between austenite and martensite phase. The entropy change ∆*S* was calculated by DSC result.

For Ni_52_Mn_25_Ga_23_, the entropy change ∆*S* due to the martensite transition is 20 J/kg·K. The magnetization change ∆*M* =11 Am^2^/kg, therefore ∆*S*/∆*M* = 1.8 T/K, and d*T*_M_/d*B* = 0.55 K/T. The experimental result of d*T*_M_/d*B* is 0.43 ± 0.1 K/T [24].

For Ni_52.5_Mn_24.5_Ga_23_, the entropy change ∆*S* is 22 J/kg·K. Magnetization change ∆*M* = 17 Am^2^/kg, therefore ∆*S*/∆*M* = 1.3 T/K, and d*T*_M_/d*B* = 0.77 K/T. The experimental result of d*T*_M_/d*B* is 0.9 ± 0.2 K/T (this work).

For Ni_52.7_Mn_24.3_Ga_23_, the entropy change ∆*S* is 26 J/kg·K. Magnetization change ∆*M* =34 Am^2^/kg, therefore ∆*S*/∆*M* =0.76 T/K, and d*T*_M_/d*B* = 1.3 K/T. The experimental result of d*T*_M_/d*B* is 1.1 ± 0.2 K/T (this work). The d*T*_M_/d*B*’s are approximately as same as that of the calculated values.

Khovailo *et al.* [34,35] discussed the correlation between the shifts in *T*_M_ for Ni_2+*x*_Mn_1−*x*_Ga (0 ≤ *x* ≤ 0.19) using theoretical calculations according to Clausius-Clapeyron formalism. The experimental values of this shift for Ni_2+*x*_Mn_1−*x*_Ga (0 ≤ *x* ≤ 0.19) are in good agreement with the theoretical calculation results. In general, in a magnetic field, the Gibbs free energy is lowered by the Zeeman energy −∆*MB*, which enhances the motive force of the martensite phase transition. Thus the *T*_M_’s of the ferromagnetic Heusler alloys are considered to have shifted in accordance with magnetic fields because high magnetic fields are favorable for ferromagnetic martensite phases.

The relationship between magnetism and *T*_M_ in magnetic fields is discussed for other Ni_2_MnGa-type Heusler alloys. Table 1 shows the spontaneous magnetizations and d*T*_M_/d*B* values of Ni_2+*x*_Mn_1−*x*_Ga, Ni_52_Mn_12.5_Fe_12.5_Ga_23_, Ni_2_Mn_0.75_Cu_0.25_Ga, Ni_2_MnGa_0.88_Cu_0.12_, and Ni_52_Mn_25_Ga_23_. As for Ni_2+*x*_Mn_1−*x*_Ga alloys, shifts in the *T*_M_ of the magnetic fields were observed by magnetization measurements [2,34,36,37]. The *T*_M_ and *T*_C_ of Ni_2_MnGa (*x* = 0) are 200 and 360 K, respectively. The region above *T*_M_ is the Ferro–A phase. A sample where *x* = 0 for Ni_2+*x*_Mn_1−*x*_Ga showed a phase transition from the Ferro–A to the Ferro–M phases at *T*_M_. A sample where *x* = 0.19 showed ferromagnetic transition and martensite transition at *T*_M_. When *x* = 0, the shift in *T*_M_ was estimated as d*T*_M_/d*B* = 0.2 K/T [20] and where *x* = 0.19, d*T*_M_/d*B* = 0.8 K/T [37]. The shift in *T*_M_ where *x* = 0.19 was higher than that for *x* = 0. The last four alloys in Table 1 show re-entrant magnetic transition. In these alloys, ferromagnetic transition occurs at the martensite transition. Below *T*_M_, the paramagnetic martensite phase (Para–M) appears. On the other hand, above *T*_M_, the austenite ferromagnetic phase (Ferro–A) appears. The ground states of these alloys are the ferromagnetic martensite phase at low temperature. Therefore, re-entrant magnetism appears. In a magnetic field, the ferromagnetic phase is more stable than the paramagnetic phase is. Therefore, *T*_M_ decreases with increasing magnetic fields while the ferromagnetic phase area increases; the sign of d*T*_M_/d*B* is negative. In these alloys, the values of d*T*_M_/d*B* are large. This means that strong magneto-structural coupling was revealed through magnetic properties and phase transitions.

### 3.4. Magneto-Structural Coupling of Ni_2_MnGa-type Heusler Alloys

Finally, we comment on the *x*–*T* phase diagram of Ni_2_MnGa-type Heusler alloys. As for the *x*–*T* phase diagram of Ni_50+*x*_Mn_27−x_Ga_23_ alloys (−25 ≤ *x* ≤ 6), the *T*_M_ increases with increasing *x*. In contrast, the *T*_C_ decreases with decreasing *x*. Kataoka *et al.* [41] explained the phase diagram of Ni_2_Mn_1−*x*_Cu*_x_*Ga (0 ≤ *x* ≤ 0.40) alloys. They conceived of the Landau-type phenomenological free energy as a function of martensitic distortion and magnetization. Their analysis showed that the bi-quadratic coupling term of martensitic distortion and magnetization, together with a higher order term, play an important role in the interplay between the martensite and ferromagnetic phases. Their calculation was based on the phenomenological free energy, shown as:

$$F_{\text{tot}}=F_{\text{ela}}+F_{\text{mag}}+F_{\text{mag}-\text{ela}}$$ (2)

where, *F*_tot_ is the total free energy; *F*_ela_, the free energy of the elastic strain *e_ij_*; *F*_mag_, the free energy of the magnetic system (including the magnetic exchange energy and the magnetocrystalline anisotropy energy) and *F*_mag−ela_, the energy of the interaction between the distortion and the magnetization. The calculated *x*–*T* phase diagram of Ni_2_Mn_1−*x*_Cu*_x_*Ga agrees well with the phase diagram, which was obtained from the experimental results. In addition, using the martensitic distortion coefficient *e*_3_, they suggested that the bi-quadratic term, $e_{3}^{2}\;M^{2}$, in *F*_mag−ela_, affects large magneto-structural coupling. ( $e_{3}=\left(2e_{zz}-e_{xx}-e_{yy}\right)/\sqrt{6}$, where, *e_xx_*, *e_yy_* and *e_zz_* are strains along *x*, *y*, and *z* axis, respectively). Thus, strong magneto-structural coupling was shown to have an important role in the magnetic properties and phase transitions of ferromagnetic shape memory alloys. Large magnetocrystalline anisotropy influences magneto-elastic coupling *F*_mag−ela_ by means of the bi-quadratic term, $e_{3}^{2}\;M^{2}$. In Ni_50−*x*_Co*_x_*Mn_31.5_Ga_18.5_ (0 ≤ *x* ≤ 9), magnetization *M* increases with the magnetic field between 338 K and 388 K [40]. The thermal hysteresis of the thermal strain also decreases at high magnetic fields. Other Heusler compounds, such as Ni_50+*x*_Mn_12.5_Fe_12.5_Ga_25−*x*_, show an *x-T* phase diagram similar to that of Ni_2_Mn_1−*x*_Cu*_x_*Ga [26]. The magnetic field-induced strain in single crystals, or magnetostriction in polycrystals, of Ni_2_MnGa, Ni-Co-Mn-Ga, and Ni-Co-Mn-In alloys also suggest a strong magneto-structural coupling. The *x*–*T* phase diagram of Ni_50+*x*_Mn_27−*x*_Ga_23_ alloys (−25 ≤ *x* ≤ 6) also shows the same characters as that of Ni_2_Mn_1−*x*_Cu*_x_*Ga (0 ≤ *x* ≤ 0.40) alloys [19]. The *x–T* phase diagram of Ni_50+*x*_Mn_27−*x*_Ga_23_ indicates a strong magneto-structural coupling for Ni_50+*x*_Mn_27−*x*_Ga_23_ alloys.

To apply this theory to our present work, further theoretical consideration is needed to apply this theory for analyzing the relationship between the martensite variant structure and the magnetic field, which is reflected by the Zeeman term, and Jahn-Teller effect, which is applied to the ferrites and perovskite compounds [42,43].

## 4. Conclusions

Thermal strain, permeability, and magnetization measurements were performed on the Heusler alloys Ni_52.5_Mn_24.5_Ga_23_ (*x* = 2.5) and Ni_52.7_Mn_24.3_Ga_23_ (*x* = 2.7).

1. Thermal strain: When cooling from the austenite phase, a steep decrease in the thermal strain was obtained because of the martensite transition. *T*_M_ and *T*_R_ increased gradually with increasing magnetic fields. For *x* = 2.5, The shift in *T*_M_ in the magnetic field was estimated as d*T*_Ms_/d*B* = 0.9 ± 0.2 K/T. For *x* = 2.7, the shift in *T*_M_ in the magnetic field was estimated as d*T*_Ms_/d*B* = 1.1 ± 0.2 K/T.
2. The d*T*_Ms_/d*B* values determined from the thermal strain results are approximately as same as the values calculated by the Clausius-Clapeyron relation.
3. For *x* = 2.5, at the Curie temperature, the *M*^4^ *vs. B/M* plot crossed the origin of the coordinate axis, and the *M*^4^ *vs. B/M* plot indicates a good linear relation around the Curie temperature *T*_C_ = 350 K. The result is in agreement with the theory by Takahashi for weak itinerant electron ferromagnets. From the magnetic moment and the gradient of *M*^4^ *vs. B/M*, we calculated the spin fluctuation parameter *T*_A_ as 1.06 × 10^4^ K, which is comparable to the value of Ni (1.76 × 10^4^ K).

## Figures and Tables

**Table 1.** Spontaneous magnetization and d*T*_M_/d*B* in Heusler Ni_2_MnGa type magnetic shape memory alloys. *M*_M_ and *M*_A_ indicate the spontaneous magnetizations in the martensite phase and austenite phases, respectively. Ferro and Para indicate the ferromagnetic and the paramagnetic phases, respectively. *T*_C_^M^ indicates the Curie temperature in the martensite phase, and *T*_C_^A^ indicates the Curie temperature in the austenite phase. The * symbols in this table (eg. *1) indicate the references as shown in Remarks column.

Sample	*M*_M_	*M*_A_	d*T*_M_/d*B*(K/T)	Remarks
Ni_2_MnGa	90 Am^2^/kg at 180 K (*1) Ferro	80 Am^2^/kg at 220 K (*1) Ferro	0.20 (*2) 0.40 ± 0.25 (*3)	*1 [2]; *2 [36]; *3 [37]
Ni_2.19_Mn_0.81_Ga	2.0 (a.u.) at 300 K Ferro	0 (a.u.) at 350 K Para	0.80 ± 0.5	[34]
Ni_52_Mn_12.5_Fe_12.5_Ga_23_	63.1 Am^2^/kg at 250 K Ferro	52.7 Am^2^/kg at 300 K Ferro	0.5	[26]
Ni_2_Mn_0.75_Cu_0.25_Ga	42.4 Am^2^/kg at 300 K Ferro	0 Am^2^/kg at 307 K Para	1.2	[26]
Ni_2_MnGa_0.88_Cu_0.12_	37.3 Am^2^/kg at 330 K Ferro	0 Am^2^/kg at 340 K Para	1.3	[38]
Ni_52_Mn_25_Ga_23_	42 Am^2^/kg at 333 K Ferro	34 Am^2^/kg at 335 K Ferro	0.43 ± 0.1	[24]
Ni_52.5_Mn_24.5_Ga_23_	39 Am^2^/kg at 338 K Ferro	22 Am^2^/kg at 343 K Ferro	0.9 ± 0.2	this work
Ni_52.7_Mn_24.3_Ga_23_	34 Am^2^/kg at 352 K Ferro	0 Am^2^/kg at 356 K Para	1.1 ± 0.2	this work
Ni_45_Co_5_Mn_36.7_In_13.3_	0 Am^2^/kg at 270 K Para	70 Am^2^/kg at 320 K Ferro	−4.3	[33]
Ni_43_Co_7_Mn_31_Ga_19_	20 Am^2^/kg at *T*_C_^M^ ≤ *T* ≤ *T*_M_ Para or weak Ferro	59.2 Am^2^/kg at *T*_M_ ≤ *T* ≤ *T*_C_^A^ Ferro	−2.95	[39]
Ni_41_Co_9_Mn_32_Ga_18_	4.0 Am^2^/kg at *T*_C_^M^ ≤ *T* ≤ *T*_M_ Para or weak Ferro	53.3 Am^2^/kg at *T*_M_ ≤ *T* ≤ *T*_C_^A^ Ferro	−2.8	[39]
Ni_41_Co_9_Mn_31.5_Ga_18.5_	12 Am^2^/kg at *T*_C_^M^ ≤ *T* = 316 K ≤ *T*_M_ Para or weak Ferro	79 Am^2^/kg at *T*_M_ ≤ *T* = 388 K ≤*T*_C_^A^ Ferro	−4.2	[40]
